# Contained Rupture of a Thoracic Aortic Aneurysm: A Rare and Life-Threatening Cause of Dysphagia

**DOI:** 10.7759/cureus.56517

**Published:** 2024-03-20

**Authors:** Marion Kestemont, Aurélien Gonze, Gaëlle Ngueke Nguimgo Gaëlle, Sofie Moorthamers, Olivier Vermylen

**Affiliations:** 1 Emergency Department, Hospital Centre EpiCura, Mons, BEL; 2 Intensive Care Unit, Erasmus Hospital, Brussels, BEL; 3 Emergency Department, Brugmann University Hospital, Brussels, BEL

**Keywords:** dysphagia at the emergency departement, rare presentation of thoracic aortic aneurysm, rare cause of dysphagia, contained rupture of aortic aneurysm, dysphagia aortica

## Abstract

Dysphagia is commonly seen in the elderly and has both benign and malignant causes. A difficulty in swallowing due to the esophageal compression by the aorta, or dysphagia aortica, is a rare entity, little described in literature. However, diagnostic error or diagnostic and treatment delays of aortic dysphagia can be fatal. Herein, we report a case of dysphagia aortica caused by the contained rupture of a descending aneurysmatic thoracic aorta, presenting at the emergency department with acute dysphagia and diffuse chest pain, successfully treated by thoracic endovascular aneurysm repair.

## Introduction

Dysphagia is subjectively defined as a difficulty in swallowing. It is a common complaint, especially in the elderly. Its prevalence is estimated at 3% in the general population and, according to some studies, may rise to 60% in nursing home residents [1,2]. However, it should not be solely attributed to normal ageing, which causes only slight and often asymptomatic abnormalities in esophageal motility [3]. The first step in the management of dysphagia is a careful and detailed history to determine whether its origin is oropharyngeal or esophageal [4].

Esophageal dysphagia has a wide range of possible etiologies, including intrinsic causes, mostly neoplastic or peptic, extrinsic causes, and esophageal motor disorders. In rare cases, severe aortic atherosclerosis or an aortic aneurysm may cause esophageal compression and aortic dysphagia [5]. Other rare causes of dysphagia with a vascular etiology are an aberrant right subclavian artery (dysphagia lusoria), a hypertrophied left atrium (dysphagia megalatriensis) or a (hemi-) azygos vein aneurysm [6,7]. 

Here, we present a rare case of aortic dysphagia caused by a contained rupture of a thoracic aortic aneurysm, which was treated by thoracic endovascular aneurysm repair (TEVAR).

## Case presentation

A 77-year-old man presented to the Emergency Department (ED) with diffuse chest pain for one week and new-onset dysphagia to both solids and liquids for 24 hours. Before arriving at the ED, he started vomiting. A single episode of hemoptysis at the ED was reported. Systemic enquiry revealed no other recent symptoms.

The patient was an active smoker and was known for arterial hypertension and an obliterated left femoropopliteal bypass of the left common femoral artery, for which he was currently receiving long-term oral acenocoumarol. He was also known for a thoracic aneurysm of the descending aorta, with measured diameters of 39 x 45 mm at the latest follow-up one year before.

At ED admission, the patient’s blood pressure was 112/48 mmHg; heart rate, 112 beats per minute; respiratory rate, 20 breaths per minute; oxygen saturation, 98% on room air; and temperature, 35.2°C. On examination, the patient was alert and oriented, hemodynamically stable and in no acute distress. Chest auscultation revealed fine crackles at the left lung base. Except for known absence of left popliteal, posterior tibial and dorsalis pedis pulses, arterial pulses were weak but palpable in all other four extremities. The abdominal exam was notable for diffuse abdominal tenderness to palpation. Neurological examination was unremarkable.

**Figure 1:**
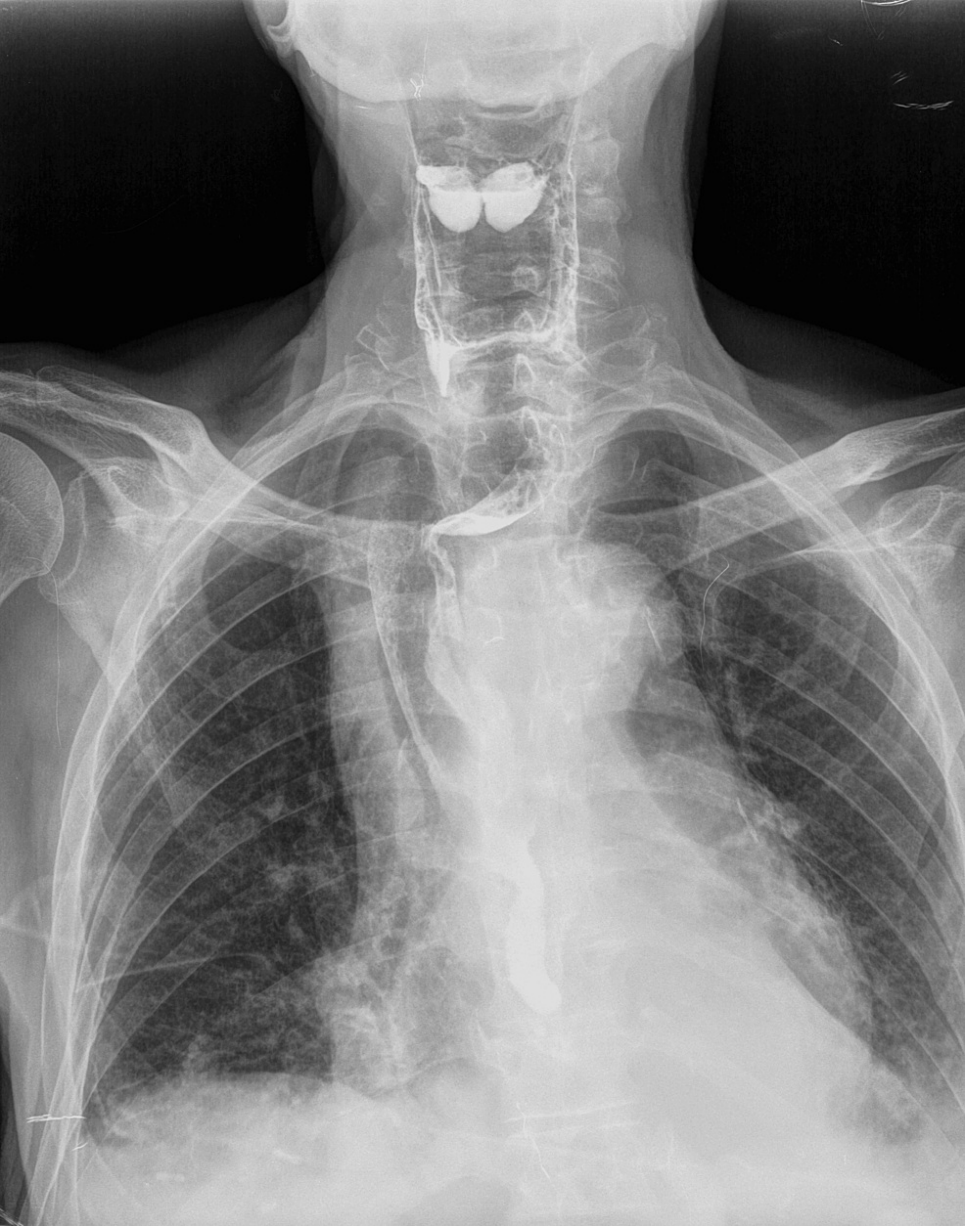
Oral gastrografin contrast study. Oral gastrografin contrast study showing mediastinal widening, esophageal narrowing at the left lateral aspect of the proximal to mid esophagus and deviation of the esophagus to the right, due to external compression by the aneurysm and its mediastinal contained hematoma.

**Figure 2:**
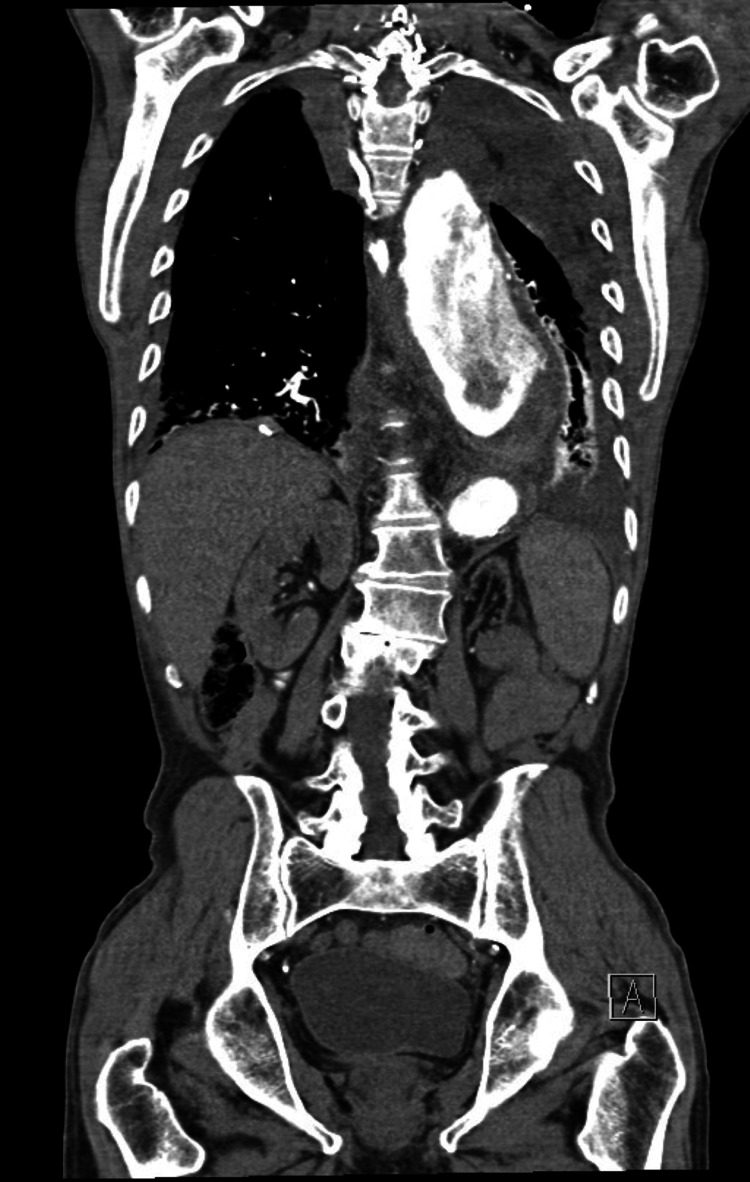
Contrast-enhanced computed tomography. Contrast-enhanced computed tomography of the chest demonstrating an acute contained ruptured thoracic aortic aneurysm with considerable compression of the esophagus by a voluminous mediastinal hematoma. Diameters of the descending aorta and the surrounding periaortic hematoma are respectively 67 x 64 mm and 78 x 70 mm.

The initial laboratory results were as follows: hemoglobin, 11.2 g/dL (normal, 13-18.0 g/dL); white blood cell count, 14.6/µL (normal, 3.5-11.0/µL); C-reactive protein (CRP), 200.1 mg/L (normal <10.0 mg/L); creatinine, 2.35 mg/dL (normal, 0.7-1.2 mg/dL); high-sensitive cardiac troponin T, 46.3 ng/L (normal, <14 ng/L); International Normalized Ratio (INR), >9 (normal, 0.95-1.31); and lactate, 2.9 mmol/L (normal 0.7-2 mmol/L). The 12-lead electrocardiogram (ECG) demonstrated sinus tachycardia (rate 111/min) with no acute ischemic or hypertrophic changes. A baseline frontal chest radiograph revealed mediastinal widening. The oral gastrografin contrast study, obtained in the ED, was highly suggestive of external compression of the proximal to mid-esophagus (Figure 1). Subsequent computed tomography angiography (CTA) of the chest and abdomen showed a large contained periaortic hematoma (78 x 70 mm) arising from the anterior side of the known aneurysm, the latter being considerably enlarged compared to the last follow-up CTA study (67 x 64 mm) (Figure 2).

After a multidisciplinary board meeting, both cardiac and vascular surgeons considered the patient a very poor surgical candidate for emergent open surgery repair and a TEVAR procedure was performed instead. Two Valiant™ thoracic stent grafts with the Captivia™ delivery system (Medtronic, Minneapolis, MN, USA) were deployed in the descending aorta by a right femoral arterial approach. Post-operative digital subtraction angiography demonstrated the correct position of both endografts with total exclusion of the aneurysm. The patient’s post-procedure course was uneventful and 48 hours after TEVAR the patient was successfully extubated and postoperative follow-up was uneventful. 

## Discussion

We present the case of a 77-year-old patient who presented to the ED with acute dysphagia. The patient’s history, with a sensation of foods and liquids sticking in the chest after swallowing begins, pointed to an esophageal origin. As discussed in the introduction, esophageal dysphagia has a wide range of etiologies; further diagnostic evaluation revealed a large contained periaortic hematoma, compressing the proximal to mid-esophagus, resulting in aortic dysphagia.

Dysphagia aortica was first described by Pape in 1932 as a difficulty in swallowing, caused by an aneurysmal, dilated or tortuous aorta compressing the esophagus [8]. It is a rare entity of which the prevalence and incidence are not well defined. According to Grimaldi et al., mean age at diagnosis is 72 years, with a male-to-female ratio of 1.1:1 [9]. Dysphagia aortica is mostly associated with an aortic aneurysm. Less commonly, it is secondary to aortic dissection, aortic pseudo-aneurysm, or a tortuous aorta [10].

In the most recent and largest review of the literature on aortic dysphagia, only five out of 69 cases of dysphagia aortica were caused by a ruptured aortic aneurysm [9]. In only one case, the aortic dysphagia was acute. In the majority of cases, aortic dysphagia is progressive, evolving over several weeks [11]. Therefore, this case represents a very rare and uncommon, but acute life-threatening presentation of aortic dysphagia, highlighting the importance of considering this pathology when a patient is presenting with acute dysphagia in the ED.

The diagnosis of aortic dysphagia is not always straightforward. There is no single test diagnostic of aortic dysphagia and no recommendations for its diagnostic work-up exist. Esophageal barium swallow may show a partial obstruction and a pulsatile movement of barium solution synchronous with aortic pulsations [12]. Esophageal manometry may reveal a high-pressure area at the site of vascular compression; esophageal endoscopy may demonstrate an extrinsic pulsatile compressive mass; and computed tomography angiography of the thoracic aorta may support the diagnosis of aortic dysphagia by demonstrating an aortic (pseudo-) aneurysm, dissection, or tortuous aorta [12,13]. However, the cornerstone in the diagnosis of dysphagia aortica remains a high clinical suspicion and the awareness among emergency physicians of this uncommon cause of dysphagia.

The treatment of aortic dysphagia depends on its clinical severity, acuteness, etiology, the patient’s comorbidities, expected survival, and the indications for surgery of a thoracic aortic aneurysm and its complications. In case of mild symptoms, conservative treatment including a change in diet towards liquid foods, management of possible hypertension and heart failure, is recommended [13]. Indications for surgical repair of a descending thoracic aortic aneurysm include: aneurysms ≥ 55 mm in diameter, rapid expansive and, as in our patient: symptomatic aneurysms [13]. According to predefined anatomical and prognostic features, an open surgical or thoracic endovascular aortic repair is selected. In poor surgical candidates the placement of a gastrostomy tube may be considered but significantly impairs survival and quality of life [13].

## Conclusions

At the moment of this writing, less than 100 cases of dysphagia aortica are described in medical literature. Although rare, esophageal compression by a pathologic aorta should not be overlooked as a possible cause of esophageal dysphagia. There is no gold standard diagnostic test for aortic dysphagia. However, thoracic computed tomography angiography should be considered early, especially in patients presenting with acute dysphagia and chest pain, cardiovascular comorbidities or a known thoracic aneurysm. Treatment of dysphagia aortica is patient-tailored. Diagnostic and management delay in dysphagia aortica are associated with a high mortality, therefore, as illustrated by this case, emergency physicians should be aware of aortic dysphagia as a rare but life-threatening cause of esophageal dysphagia.
